# A multi-output network with U-net enhanced class activation map and robust classification performance for medical imaging analysis

**DOI:** 10.1007/s44163-022-00045-1

**Published:** 2023-01-03

**Authors:** Jaiden Xuan Schraut, Leon Liu, Jonathan Gong, Yiqiao Yin

**Affiliations:** 1 University of Michigan–Ann Arbor, Ann Arbor, USA; 2 Columbia University, New York, USA

**Keywords:** Multi-output network model, U-net, Class activation map, Image classification, Medical imaging analysis

## Abstract

Computer vision in medical diagnosis has achieved a high level of success in diagnosing diseases with high accuracy. However, conventional classifiers that produce an image-to-label result provide insufficient information for medical professionals to judge and raise concerns over the trust and reliability of a model with results that cannot be explained. To gain local insight of cancerous regions, separate tasks such as imaging segmentation needs to be implemented to aid the doctors in treating patients which doubles the training time and costs which renders the diagnosis system inefficient and difficult to be accepted by the public. To tackle this issue and drive the AI-first medical solutions further, this paper proposes a multi-output network which follows a U-Net architecture for image segmentation output and features an additional CNN module for auxiliary classification output. Class Activation Maps or CAMs are a method of providing insight into a convolutional neural network’s feature maps that lead to its classification but in the case of lung diseases, the region of interest is enhanced by U-net assisted Class Activation Mapping (CAM) visualization. Therefore, our proposed model combines image segmentation models and classifiers to crop out only the lung region of a chest X-ray’s class activation map to provide a visualization that improves the explainability and can generate classification results simultaneously which builds trust for AI-led diagnosis system. The proposed U-Net model achieves 97.72% accuracy and a dice coefficient of 0.9691 on a testing data from the COVID-QU-Ex Dataset which includes both diseased and healthy lungs.

## Introduction

Medical imaging is a vital and necessary tool for understanding the structure of the human body in diagnosis, treatment, research, and clinical contexts [1, 2]. Historically, chest X-ray has been the most utilized radiological examination. In 2006, approximately 129 million chest x-rays were obtained in the United States alone [3]. The high demand can be attributed to the low cost, widespread availability, and the non-invasive properties of X-rays as compared to alternative imaging tests, such as a diagnostic ultrasound, computed tomography (CT), or magnetic resonance imaging (MRI) [4]. As such, the chest x-ray is typically the first medical image captured and remains vital in prognosis and treatment thereafter [5]. To accurately diagnose prevalent respiratory disease, lung segmentation of chest X-rays is necessary. Medical imaging segmentation plays a critical role in analyzing medical imaging through feature extraction, a process which partitions an image by identifying homogeneous properties [6]. A region may be divided by brightness, texture, and color in the characteristics of adjacent pixels. Pixels in images take values between 0 and 255 in greyscale.

Radiologists perform manual segmentation, a time-consuming and arduous task, which suffers from high-observer variability due to conflicting interpretation [7, 8]. In lung segmentation, it is very difficult to identify small or subtle abnormalities, or to precisely differentiate between pathological patterns of diseases [9]. Other work has investigated the Chest X-ray segmentation against the SOTA methods [10]. Older chest x-ray images were sized 128 by 128, with modern imaging having significantly higher resolution. Even at this relatively low resolution, (128 * 128 = 16,384 pixels) it is considered high dimensional data for human eyes to precisely observe. Differentiation of non-COVID viral pneumonia and COVID-19, both similar in appearance on chest X-ray, is important for determining appropriate treatment [11]. The inherent difficulty in human interpretation of chest X-ray analysis has led researchers to pursue automated segmentation algorithms for this purpose.

Similarly, deep convolutional neural networks (CNN) [12] have demonstrated effective image classification, image segmentation, and semantic segmentation, a process of classifying each individual pixel of an image [13–15]. Fully Convolutional Networks (FCN) [15], a type of CNN, have been used extensively in modern semantic segmentation algorithms. Existing methods in [16] have developed Grad-CAM for imaging analysis and specifically at identifying regions. In [17], the researchers advocate for U-Net, an encoder-decoder network of FCN for biomedical image segmentation. Since its introduction, the U-Net architecture has demonstrated significant success, thus, recent studies have focused on further developing and applying this architecture rather than proposing new architectures and concepts [18]. In the future, it is in high demand to provide comprehensive medical image analysis may require simultaneous segmentation and classification to reduce doctor’s workloads, yet such methodology does not exist in the literature thus far.

This paper proposes a modification to the existing U-Net model which accomplishes the task of classification and segmentation concurrently. The major contributions of the paper are as follows:

1. We have investigated a large set of Chest X-ray images including 4 different classes with distinguishable masks to locate the region of cancerous areas.
2. A multi-output hybrid model involving segmentation and classification is proposed to detect the type of lung disease (pneumonia or COVID-19) and their cancerous region simultaneously.
3. U-net enhanced visualization using Class Activation Mapping (CAM) is used to further annotate the pixel-level activation that is implemented to generate the classification results.

## Proposed methods

In recent years, the U-Net architecture [17] has been recognized as one of the leading methods in medical image segmentation. The U-Net architecture builds on the Fully Convolutional Network architecture, by implementing upsampling operators in the contracting network, and symmetry in the contracting and expansive paths. The name comes from the U-shaped architecture represented by the contracting and expansive paths. Upsampling operators increase the output resolution to allow for precise segmentation. This paper proposes to update “upsampling” with “Conv2DTranspose” action. The symmetry within the model supports an overlap-tile strategy, which allows the network to learn efficiently even with little training data, a persistent issue in the field of medical imaging. U-Net also supports multi-scale prediction and deep supervision, as U-Net uses skip connection across the model, more low-level features have presence in the segmented output. U-Net was chosen both for the task of lung segmentation, and for the ease of implementation in the novelty of this model, an auxiliary classification output.

The innovation here is the integration of a CNN [12] to the latent layer of the U-Net. It can be said that this model shows similarity to the Siamese network architecture [19] as both the U-Net and CNN take advantage of the contracting path for feature extraction. While the model only takes one input, it’s worth mentioning that the model utilizes identical weights and biases to generate output. A traditional CNN is composed of a similar feature extraction module, a classification module and a probabilistic distribution to display output. The classification element of the multi-output approach might achieve a potential benefit from backpropagation through the segmentation element. The contracting path serves both the U-Net and the CNN, similar to the concept of a siamese [19] network.

**Fig. 1:**
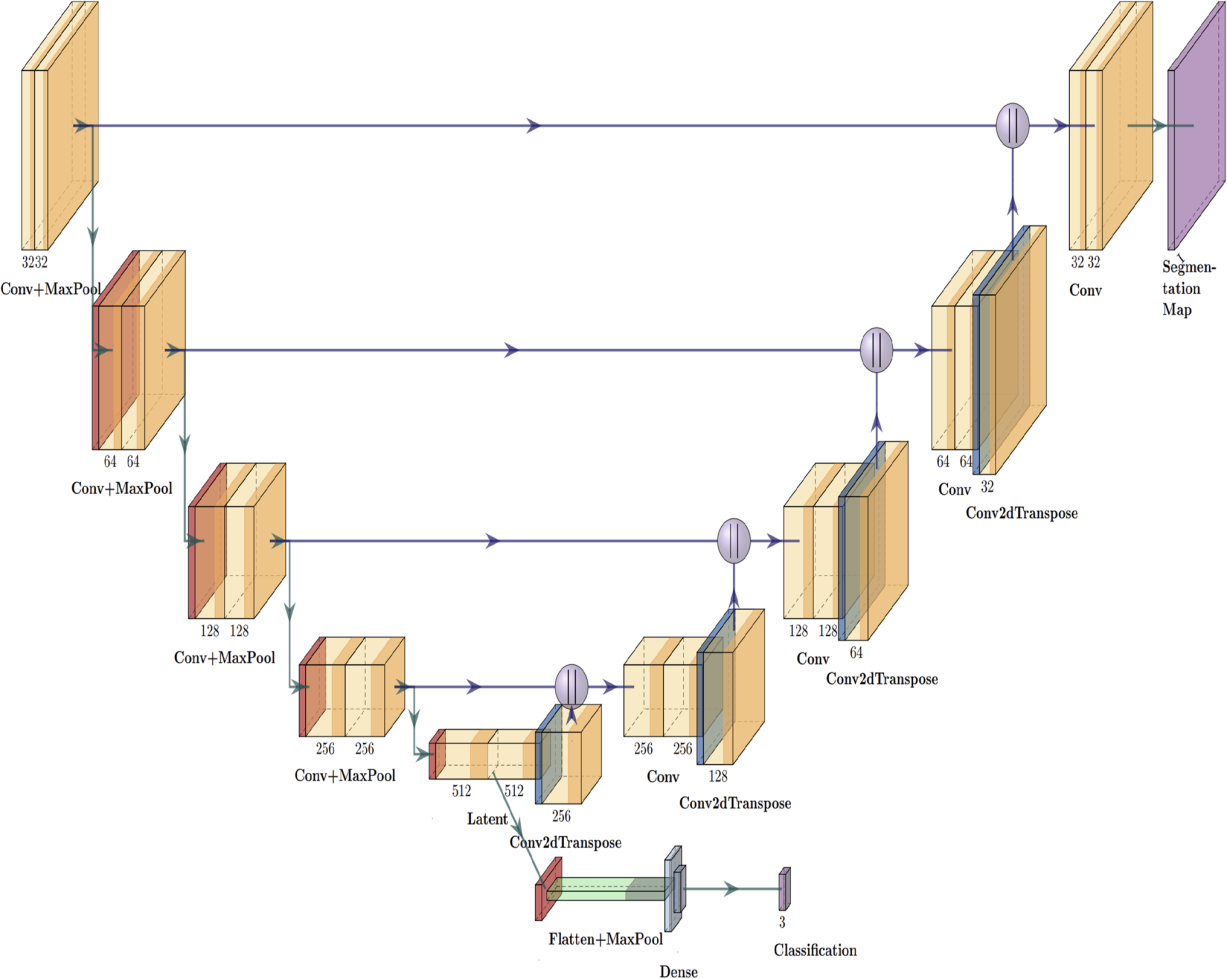
Executive diagram of the proposed model. This figure shows the proposed model for this paper. The proposed model uses U-net as the backbone. Each successive layer has a stride of 2 for downsampling, which doubles the number of feature channels. At the latent layer, the model generates the features and feeds them into max pooling layers, a flatten layer, and 3 dense layers for classification, creating an auxiliary branch. The decoding path of the model follows U-net structure and the symmetry principle. It uses Conv2DTranspose operation and the structure behaves like an inverse convolutional layer with a 2 by 2 stride level

The proposed network architecture is shown in Fig. 1. Much like the standard U-Net model, the contracting path is composed of multiple convolutional blocks each accompanied by a $2 \times 2$ max pooling layer. Each successive layer has stride 2 for downsampling, which doubles the number of feature channels. The stride hyperparameter comes from the original U-Net architecture and a stride of 2 reduces each feature maps dimension in half [17]. At the latent layer, the model splits into a max pooling layer, a flatten layer, and 3 dense layers for classification. All dense layers have ReLU activation function (3), with the exception of the final dense layer. The nature of the classification problem requires the final dense layer to be a softmax activation function, as there are multiple classes. On the expansive path of the U-Net model, following the principles of symmetry, an equal amount of convolutional blocks accompanied by a $2 \times 2$ Conv2dTranspose layer, with stride 2, followed by a concatenate layer. The Conv2dTranspose layer behaves similarly to an inverse convolutional layer with a $2 \times 2$ stride. The layer has an upsampling effect while interpreting the input data to assure detail. The concatenate layer combines the cropped feature map from the opposite convolutional block in the contracting path. This is performed as edge pixel data is lost in the process. The final layer is a $1 \times 1$ convolutional layer connect to the component feature vectors to relevant classes. Each convolutional block consists of two $3 \times 3$ unpadded convolutional layers, two Batch Normalization layers, and two ReLU activation layers for the contracting path, and two Leaky ReLU activation layers for the expansive path. Batch Normalization is used in this model to further enhance model performance by stabilizing the learning process through normalizing activation vectors without compromising on training convergence. The U-Net based multi-output architecture was realized through the Tensorflow and Keras Python libraries.

The proposed network has an auxiliary branch that produces classification results. Classes can categorize the region to isolate a mask of the desired object and exclude everything else in a two-class system. For diagnosis, this mask can be categorical and represented in 0s and 1s for a two-class classification. By multiplying a segmentation mask of 0s and 1s over the original image, only the 1s appear in the final image and generate a cropped image of the region of interest.

The proposed method for explaining diagnosis of lung diseases does not apply image segmentation to isolate the deleterious regions but instead crops out the lungs on chest X-rays in combination with a more detailed heatmap that is generated by dissecting a separate CNN model. However, image segmentation is still critical to separating the lungs from the remaining area which is not involved with the diagnosis.

This proposed incorporates a Gradient-weighted Class Activation Mapping (Grad-CAM) as a heatmap to explain a CNN’s classification.

The heatmap is colored with warmer colors indicating the local areas where the disease is and is based upon the features that cause the classifier’s prediction. The heatmap is created by extracting the feature maps from the last convolutional layer from convolutional neural networks to indicate which areas of the image produce a classification of a particular disease. More specifically, the heatmap is the weighted average of each feature map in the final convolutional layer based on its weights in connection to a final specific class probability in the softmax layer [20]. Convolutional neural networks detect more general details in shallower convolutional layers but become increasingly discriminative towards a class or object deeper into the network so the last convolutional layer is selected to generate a Gradient-weighted Class Activation Mapping (Grad-CAM) heatmap [20, 21]. This technique of generated a cropped chest X-ray through image segmentation with a U-Net is combined with a Grad-CAM heatmap to crop out the lungs and highlight colored areas that indicate where the disease is detected [17].

## Application

The dataset used to train both the CNN classifier models and U-Net image segmentation model is the COVID-19 Radiography Database found on Kaggle, based on the radiography dataset COVID-QU-Ex Dataset professionally labelled by researchers at Qatar University with chest X-rays of 4 classes: Normal, COVID-19, Viral Pneumonia, and Lung Opacity (generic Non-COVID, Non-Viral lung infection) [22, 23]. There are 10,701 Normal, 3616 COVID-19 chest x-rays, 6012 Lung Opacity chest x-rays, and 1345 Viral Pneumonia chest x-rays. For each image, there is a professionally annotated Ground Truth mask that represents the actual lung region and its label. A link of the source can be downloaded from Kaggle [24].

### Training

When training the U-Net model, the chest X-rays from all classes were compiled as 21,165 images for the input and the corresponding lung masks were compiled from all classes as the output. The U-Net was not used for classification and only for segmentation of the lung region so keeping class labels was not necessary. The data was then split into training, testing, and validation. 20% of the dataset, 4233 images, was selected randomly as testing data which the model would be evaluated with but not trained on. The random selection was seeded such that every model would have the same testing data. The remaining 80%, 16,932 images were not all used for training to conserve training time. Batches of 300 randomly selected images were selected from the training dataset and then 10% of the unused training images were selected as validation data. Batches were selected 6 times for each U-Net to train upon in 6 training sessions. The training and testing sample size for U-net segmentation task is presented in Table 1. For the CNN classifier, the training and testing sample size is discussed in Table 2.

**Table 1:** U-Net train-test split

Training	Testing	Validation
1800	4233	1693

The training and testing data are randomly selected. 20% of the dataset is reserved for testing data. 10% of the remaining (8% of the original) images are used for validation. The remaining data is selected in batches of 300 to train for 200 epochs 6 times for a total of 1800 training images

**Table 2:** Classification train-test split

Training	Testing	Validation
10,000	4233	693

The training and testing data are randomly selected. 20% of the dataset is reserved for testing data. From the remaining data, 10,000 images were randomly selected for the training data. Then, 693 images (10% of the remaining 6932 images) were reserved as validation data

For training the classification models, the input chest X-rays were compiled together, and the output was the corresponding labels of each input feature. The dataset was then split into training, validation, and testing. Testing data consisted of a randomly selected 20% of the dataset, 4233 images, which would not be used for training the classification models. The random selection was seeded such that every model would have the same testing data. The remaining 80%, 16,932 images were not all used for training to conserve training time. 10,000 images were randomly selected as training data and the split was seeded so each model would receive the same training data. After that the remaining unused 6,932 images were split for 10% (693 images) to become validation data that would evaluate the model from epoch to epoch while training.

All input chest X-ray images are rescaled if it is formatted in the RGB 0-255 value scale to instead range from 0 to 1. Rescaling is done using the Eq. 1 on the entire image array:

1 $$ x_\text {rescaled} = \frac{x}{x_{\max }} $$

### Loss functions

The various U-Net models with different hyperparameters for encoding blocks and filters were trained on the randomly selected batches of 300 images for 200 epochs 6 times for a total of 1200 epochs. The training results are featured in Table 3. For each model, checkpoints were used to save the best model weights based on loss during training. During training, sparse categorical cross-entropy was as the loss function for training. The categorical cross-entropy formula is defined in the following, Eq. 2,

2 $$\mathcal {L}(y, \hat{y}) = \frac{1}{n} \sum _{i=1}^n y_i \log (\hat{y}_i) + (1 - y_i) \log (1 - \hat{y}_i) $$

**Fig. 2:**
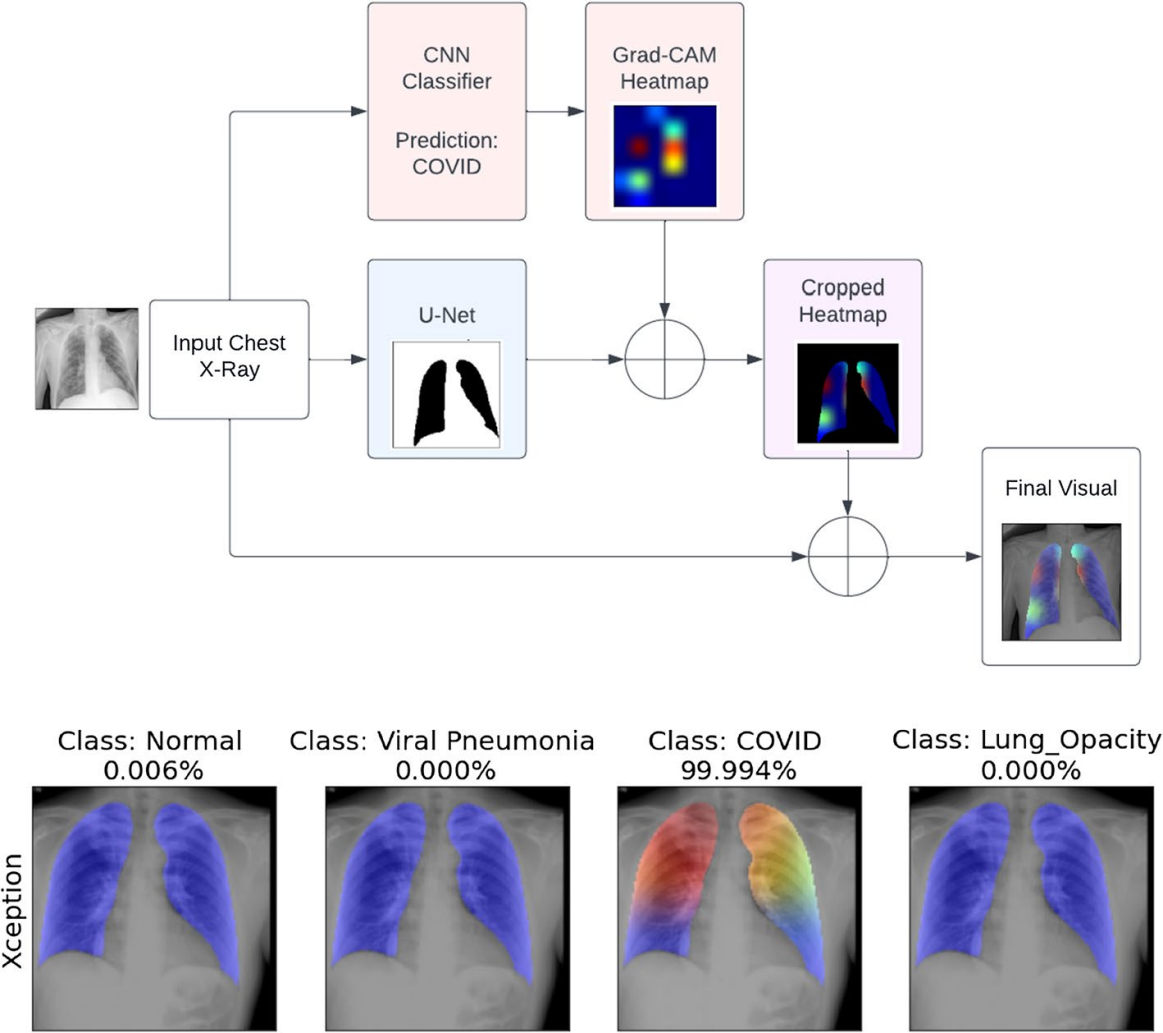
Proposed workflow of the full visualization process. This diagram shows the workflow to use U-net and Class Activation Mapping (CAM) to produce highlighted heatmaps. Producing a final visualization involves inputting the chest x-ray through a U-Net image-to-image model to generate a class-discriminatory mask that distinguishes the lung area. The chest x-ray is also inputted through a CNN that classifies the image and its feature maps are extracted produce a Grad-CAM heatmap that indicates with warm colors which areas led to the classification. The final visualization involves cropping the heatmap with the mask and then overlaying it on top of the original chest x-ray

**Fig. 3:**
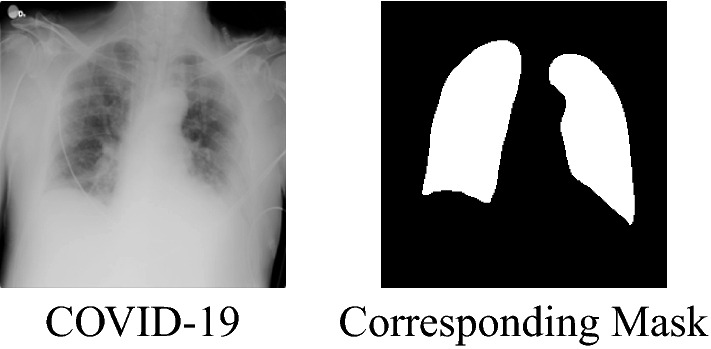
Example chest X-ray. This is a sample X-ray from training data. The plot on the right is a 2D array with values of 1 representing lungs and 0 representing not lungs. This allows the mask to be multiplied with the original image element wise to crop out the lungs without the heart in the middle or the remaining X-ray

Sparse categorical cross-entropy just uses integer class labels rather than one-hot encoded labels. After each model was trained it would be evaluated on the testing data of 4233 images and the predicted mask results would be evaluated against the ground truth masks with conventionally-used dice loss, sparse categorical cross entropy loss, and pixel-wise accuracy (Figs. 2, 3).

**Table 3:** Segmentation and classification results

Model	Segm.	Classi.	Time
	Dice Coef.	Accuracy	
U-net (benchmark^a^)	0.86	–	
U-net (our repl., alone)	0.97	0.98	11 min (100 ep)
ConvNet (benchmark^b^)	–	0.86–0.95	
ConvNet (our repl., alone)	–	0.95	2 min (100 ep)
Multi-output (proposed	0.865	0.95	11 min (100 ep)

Model	100 epochs	Latency	
U-net (alone)	11 min 39 s	0.00517 s	
CNN (alone)	2 min 37 s	0.00331 s	
Combined	14 min 16 s	0.00848 s	
Proposed model	11 min 44 s	0.01017 s	

The results for segmentation (use Dice Coefficient) and classification (use Accuracy) are presented below. Time consumption for 100 epochs is also presented

^a^Refers to [1]

^b^Refers to [26–30]

Dice loss is the complement of the dice coefficient (see Eq. 4) as it is formally written below.

3 $$ \text {Dice}\; \text{Loss} = 1 - \text {DSC} $$

Dice coefficient is

4 $$ \text {DSC} = \frac{2 \text { intersection}} {\text{union} + \text {intersection}} = \frac{2\text {TP}}{2\text {TP}+\text {FN}+\text {FP}} $$

where TP refers to true positives, FN refers to false negatives and FP refers to false positives. Sparse categorical cross entropy loss is the same equation used to train the model (see Eq. 2).

### Performance measure metrics

**Fig. 4:**
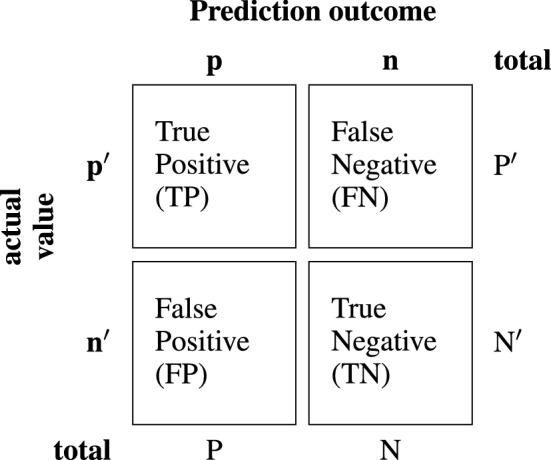
Confusion matrix. A confusion matrix describes the performance of the model indicating how well it predicted each classification according to the actual label. In this matrix, TP refers to true positives, FN refers to false negatives and FP refers to false positives

The Intersection over Union (IoU) (Eq. 5), commonly known as the Jaccard index, is the area of overlap between the predicted segmentation divided by the area of union between the predicted segmentation and the ground truth. For application in image segmentation, the mean IoU of the image is calculated by taking the IoU of each class and averaging them. The formula for IoU is formally defined below

5 $$ \mathcal {J}(A,B) = \frac{|A \cap B|}{|A \cup B|} $$

where *A* and *B* are two sets (Fig. 4).

### Activation functions

To improve the performance of the model, the effects of several activation functions were compared, prompted by the work of [25]. The authors investigated the differences in performance of activation functions in image analysis. The study concluded that in the task of medical image analysis, there may be marginal improvement in results based on selected activation function. In response to their findings, the model was evaluated separately with each of these functions in image segmentation. The activation functions were implemented in the decoder block of the model.

**Fig. 5:**
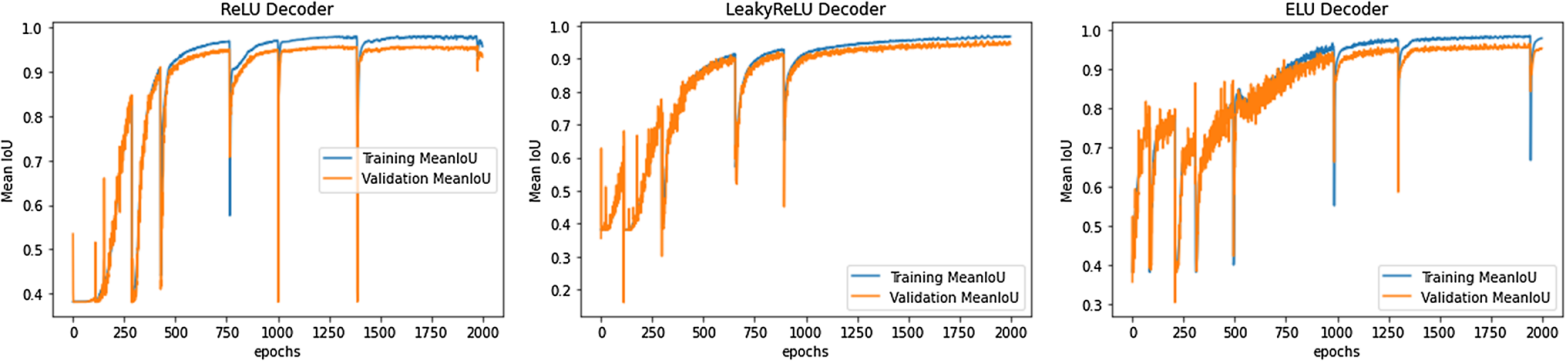
Comparative activation performance. This figure presents the training and validation paths under different activation performance

The ReLU activation function takes an input and allows the information to pass through if it is non-negative. Updated versions of ReLU can also be used. Leaky ReLU is one of its upgrades and it lets information passes through with a scaling factor of $\alpha$. In addition, exponential scaling can also be used. The results of these experiments is in Fig. 5.

6 $$  \text {ReLU}(z) = \max (z, 0) $$

7 $$ \text {Leaky ReLU}(z) = \max (\alpha z, z)$$

where $\alpha$ is a tuning parameter.

8 $$\text {ELU}(z) = \max (z, \alpha (e^z - 1)) $$

The standard rectified linear unit (see Eq. 6) activation function takes an input and allows the information to pass through if it is non-negative. Updated versions of ReLU can also be used. Leaky rectified linear unit (see Eq. 7) is one of its versions and it lets information passes through with a scaling factor of $\alpha$. Exponential linear unit (see Eq. 8) is another version of ReLU with exponential scaling. ReLU is the predominant activation function for training deep and traditional neural networks. The ReLU activation function accelerates the training rate of deep and traditional neural networks compared to conventional activation functions as the derivative of ReLU is 1 for a positive input. The network saves time for computing error metrics in the process of training. As well as increasing computational efficiency, ReLU does not prompt the vanishing gradient problem when the model increases layers. The vanishing gradient problem occurs when the partial derivative of the loss function approaches zero. In neural networks that rely on backpropogation or gradient-based learning, the partial gradient effects the weights proportional to the value of itself. A value approaching zero will become so insignificant that the model may prevent the weight from changing its value, stopping the model from training altogether. Hyberbolic tangent and sigmoid activation functions are known to be susceptible to this problem as well. Leaky ReLU is a modification of ReLU, producing small output values given a negative input in comparison to a value of zero given by the ReLU function in the same scenario. This modification prevents the dying ReLU problem, where a neuron may learn a large negative bias and continually output the same value. This neuron is ’dead,’ and will now have no future effect on the model, as it is improbable to learn when the function gradient is at 0. The nonzero output value given a negative input gives a ’dead’ neuron a chance to become active. The ELU activation function is an identity function for non-negative inputs, like ReLU. ELU differs from ReLU due to an $\alpha$ constant which determines function smoothness for negative inputs. ELU does not suffer from the dying neuron problem and the vanishing gradient problem, while generalizing better. ELU tends to have a comparatively faster convergence time than ReLU, though it is slower to compute due to the non-linearity calculations necessary for negative inputs.

Since the prediction of a mask or a class can be coded to ones and zeros, the prediction is based on the true positives (TP), true negatives (TN), false positives (FP) and false negatives (FN), the accuracy can be formally defined using the following formula

9 $$ \text {Accuracy} = \frac{\text {TP}+\text {TN}}{\text {TP}+\text {TN}+\text {FP}+\text {FN}} $$

The testing results of the various U-Net models (see Table 4) demonstrated a positive correlation between the number of encoding and decoding blocks and accuracy as well as lower dice loss, sparse categorical cross-entropy loss. Both loss-functions were positively correlated with each other and inversely proportional to accuracy as loss functions. Out of the tested parameters, the best number of encoding and decoding blocks based on the metrics used was 5. The other hyper-parameter tested was the number of filters in the first encoding block which would be doubled with each block and then halved with each decoding block. The number of filters reduced accuracy when increased. Out of the tested parameters, the best number of filters was 16 in the first encoding block. The best model U-Net (5E16F) had an accuracy of 97.61% and has a dice loss of 0.0335. Their source images could not be found so a similar dataset of diseased and healthy lungs was used with similar classes that contained blurrier lung sections from disease. The proposed U-Net (5E16F) has improved dice loss and accuracy in comparison to these similarly complex models even though the proposed model viewed 1800 images in comparison to 2785 images. For the computational time, the proposed model performs more accurately and is more resource efficient.

**Table 4:** Dice loss, sparse-cross-entropy loss and accuracy of U-Net models image segmentation for lungs on chest X-rays from testing data with different filter and encoding block hyperparameters

Model variants	Dice loss	SCCE loss	Accuracy
U-net (3L16F)	0.0338	0.0808	0.9758
U-net (3L32F)	0.0381	0.1084	0.9739
U-net (3L64F)	0.0404	0.1508	0.9727
U-net (4L16F)	0.0354	0.0666	0.9751
U-net (4L32F)	0.0335	0.0809	0.9758
U-net (4L64F)	0.0395	0.1725	0.9732
U-Net (5E16F)	**0.0309**	**0.0745**	**0.9772**
U-net (5L32F)	0.0414	0.0985	0.9722
U-net (5L64F)	0.0435	0.0766	0.9714
U-Net*1	0.0471	NA	0.9771
U-Net*2	0.0418	NA	0.9767

U-net variants	Dice loss	SCCE loss	Accuracy
U-net	0.0311	0.0748	0.9649
Dense U-net	0.0402	0.0802	0.9642
Res U-net	0.0437	0.0896	0.9701
Attention U-net	0.0422	0.0841	0.9617
Proposed U-net	**0.0309**	**0.0745**	**0.9772**

Each model was trained for 200 epochs with a batch size 32 on 6 300 training image subsets. The number before “E” indicates the number of encoding blocks and the number of decoding blocks. The number before “F” indicates the number of filters per convolutional layer. The testing data size is 4322 images. The benchmarks are U-Net*1 and U-Net*2. U-Net*1 is U-net architecture + Efficientnet-b4 encoder method U-Net*2 is U-net architecture + Efficientnet-b4 encoder + LeakyReLU method [31, 32]. These benchmarks do not have values for sparse categorical cross-entropy loss. Additional variants of U-net such as Dense U-net, Res U-net, and Attention U-net are tested against the same held out test and the results are presented in this table

Bold values represent the best performance of that column. For model variants, U-net (5E16F) has the best performance because it has the lowest Dice loss, the lowest SCCE loss, and the highest accuracy. For comparisons amongst different U-net variants, the proposed U-net has the lowest Dice loss, the lowest SCCE loss, and the highest accuracy

Moreover, classification results using transfer learning are also presented in our work and these results are shown in Table 5. Although the best model performs with an overall 0.9513 accuracy this is the performance over all the 4 class types. The model is better at classifying Normal lungs with 97% recall and COVID with 97% recall while having only 90% recall for lung opacity which may be because of the variety of conditions that fall under lung opacity or the distribution of classes in random sampling. In Table 4, the standard deviation of the dice loss is 0.00471 and that of the accuracy is 0.002.

**Table 5:** Accuracy of various transfer models on 4 classes: normal, COVID, viral pneumonia, lung opacity

Model	Test accuracy
VGG16	0.9256
VGG19	0.9284
ResNet50	0.9440
ResNet101	0.8774
ResNet152	0.9310
ResNet50V2	0.8942
ResNet101V2	0.9391
ResNet152V2	0.9334
Xception	0.9513
InceptionResNetV2	0.9161
DenseNet121	0.9452
DenseNet169	0.9457
DenseNet201	0.9317

The testing data size is 4322 images. Each model was trained on the same 10,000 images for 100 epochs. The best model by accuracy is Xception with 95.13% accuracy

**Fig. 6:**
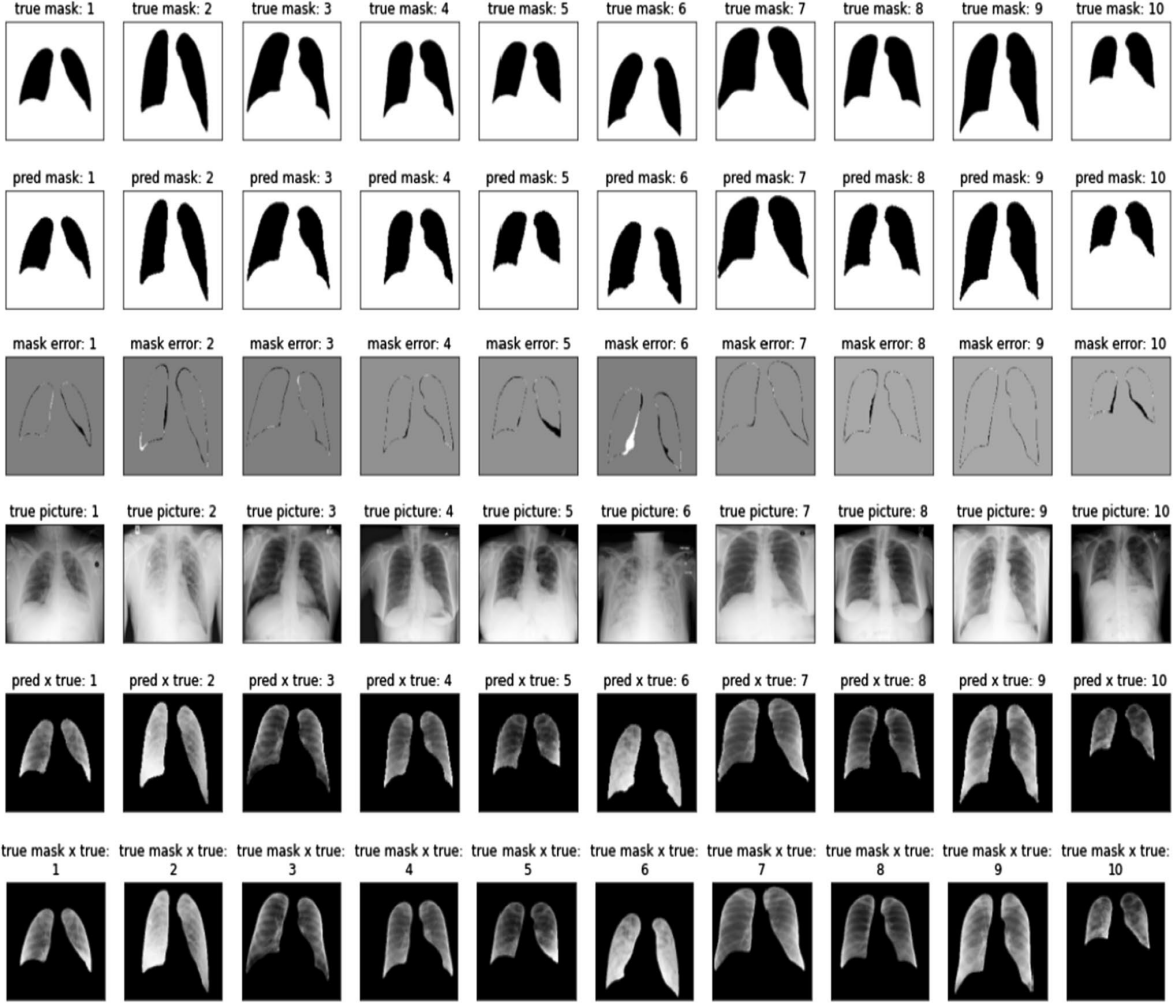
Visualization results from segmentation. The figure presents the true masks, predicted masks, errors between true and predicted masks, the original images, cropped images using predicted masks, and cropped images using true masks. These samples are selected randomly from the testing data

Visualization is presented in Fig. 6. The mask error is the difference between the ground truth mask and predicted mask where black indicates missing, white indicates additional, and gray indicates no error. As demonstrated by the model’s high accuracy the masks visually appear to be very similar with errors only around the borders where the region blurs. Even in pictures with very high lung opacity like column 2 and column 6, the model can distinguish the lungs. The error in column 6 reveals how the model performs slightly worse on chest X-rays where the lungs are cloudy from disease.

## Conclusion

This paper has proposed a model architecture with concurrent image segmentation and classification output. The proposed method achieves a Dice Coefficient of 0.9691 and an accuracy score of 0.95 on the COVID-19 Radiography database for evaluating the segmentation and classification modules, respectively. Though the concept of a concurrent segmentation and classification architecture in medical imaging is relatively novel, the applications of acquiring deep learning technology can be further exploited to other medical imaging techniques such as CT scan, MRI. Moreover, attempts can even be made to extend this analysis to ultrasound diagnostic, and many other applications in the digital pathology.

## Data Availability

The datasets generated during and/or analysed during the current study are available from the corresponding author on reasonable request.
